# Development and Qualification of an Antigen Integrity Assay for a *Plasmodium falciparum* Malaria Transmission Blocking Vaccine Candidate, Pfs230

**DOI:** 10.3390/vaccines10101628

**Published:** 2022-09-28

**Authors:** Kazutoyo Miura, Thao P. Pham, Shwu-Maan Lee, Jordan Plieskatt, Ababacar Diouf, Issaka Sagara, Camila H. Coelho, Patrick E. Duffy, Yimin Wu, Carole A. Long

**Affiliations:** 1 Laboratory of Malaria and Vector Research, National Institute of Allergy and Infectious Diseases, National Institutes of Health, Rockville, MD 20852, USA; 2 PATH’s Malaria Vaccine Initiative (MVI), Washington, DC 20001, USA; 3 Malaria Research and Training Centre, University of Science, Techniques and Technologies, Bamako 1805, Mali; 4 Laboratory of Malaria Immunology and Vaccinology, National Institute of Allergy and Infectious Diseases, National Institutes of Health, Bethesda, MD 20814, USA

**Keywords:** subunit vaccine, integrity, potency, assay qualification, malaria, transmission-blocking vaccine, Pfs230

## Abstract

During development of a subunit vaccine, monitoring integrity of the recombinant protein for process development and quality control is critical. Pfs230 is a leading malaria transmission blocking vaccine candidate and the first to reach a Phase 2 clinical trial. The Pfs230 protein is expressed on the surface of gametes, and plays an important role in male fertility. While the potency of Pfs230 protein can be determined by a standard membrane-feeding assay (SMFA) using antibodies from immunized subjects, the precision of a general in vivo potency study is known to be poor and is also time-consuming. Therefore, using a well-characterized Pfs230 recombinant protein and two human anti-Pfs230 monoclonal antibodies (mAbs), which have functional activity judged by SMFA, a sandwich ELISA-based in vitro potency assay, called the Antigen Integrity Assay (AIA), was developed. Multiple validation parameters of AIA were evaluated to qualify the assay following International Conference on Harmonization (ICH) Q2(R1) guidelines. The AIA is a high throughput assay and demonstrated excellent precision (3.2 and 5.4% coefficients of variance for intra- and inter-assay variability, respectively) and high sensitivity (>12% impurity in a sample can be detected). General methodologies and the approach to assay validation described herein are amenable to any subunit vaccine as long as more than two functional, non-competing mAbs are available. Thus, this study supports future subunit vaccine development.

## 1. Introduction

Malaria remains one of the biggest health problems globally, and the World Health Organization (WHO) estimated 627,000 malaria-related deaths, mostly in children aged under 5 years, in 2020 [1]. Therefore, in addition to the expansion of existing anti-malarial control measures, it is critical to develop new tools, such as vaccines, to fight malaria. In October 2021, the first malaria vaccine, RTS,S/AS01 (RTS,S), which is a pre-erythrocytic vaccine (PEV), was recommended by WHO to prevent *Plasmodium falciparum* malaria in children living in moderate to high transmission areas [1]. PEV is designed to block sporozoite invasion to human hepatocytes and/or inhibit growth of parasites in the hepatocytes, thus it can prevent any symptoms caused by malaria infection. While this was a major milestone, further improvements in terms of the efficacy and duration of protection, and/or a combination with other intervention(s) are likely to be required for elimination of malaria [2].

Transmission-blocking vaccines (TBVs) target a different life stage of malaria parasites, and are designed to induce antibodies in human hosts that inhibit parasite development in the mosquito bloodmeal [3]. Using a mouse malaria model, a synergistic effect, better than an additive effect, has been shown with a combination of PEV and TBV [4], indicating a TBV could be a strong additional tool to malaria control and eradication. Pfs230 is one of the leading TBV candidates, and the first to reach a Phase 2 clinical trial as a recombinant protein sub-fragment conjugated with *Pseudomonas aeruginosa* ExoProtein A (ClinicalTrial.gov Identifier: NCT03917654). While the ultimate method to determine efficacy of a TBV is to measure reduction of malaria transmission in a field site by a cluster randomized trial, such studies are large, complex and costly, and cannot be used in early stages of TBV development. Thus, the current gold standard method to measure efficacy of vaccine-induced antibodies (either from animals or humans) in early stages of TBV development is the standard membrane-feeding assay (SMFA) or the direct membrane-feeding assay (DMFA) [3]. In SMFA/DMFA, control or test antibodies are mixed with either cultured malaria (for SMFA) or blood from a malaria-infected human (for DMFA) and fed to mosquitoes. Approximately a week after the feed, the mosquitoes are dissected, and the reduction in parasite oocyst density in the mosquito midguts (called transmission-reducing activity (TRA)) or reduction in the proportion of infected mosquitoes (called transmission-blocking activity (TBA)) is determined by comparing the control and test groups.

During development of a subunit vaccine, the integrity of the recombinant protein must be monitored for process development and quality control of intermediate products. Furthermore, lot-to-lot consistency and stability of drug substance or drug product must be assessed. Multiple biophysical and biochemical methods, such as western blot, high-performance liquid chromatography (HPLC), circular dichroism, static light scattering, and dynamic light scattering, are commonly used for the quality control of vaccines, but a potency assay is another key evaluation [5,6]. In the case of a malaria TBV, based on the mode of action, an in vivo immunization study (often in mice) that assesses antisera or induced antibodies by SMFA is an acceptable potency assay. However, antibody titers in animal studies can fluctuate significantly even when the same batch of vaccine is utilized for immunization [6]. For example, in the case of Diphtheria, Tetanus and acellular Pertussis (DTaP) vaccine, the coefficients of variance (CV) in antibody titers against pertussis antigen were reported as high as 131.7% in mouse immunization studies [7]. In addition, precision in SMFA is poor when activity of a test antibody is low [8]. These characteristics, coupled with the duration to conduct such testing and limited throughput, lead to the need for additional in vitro biochemical methods that may be a surrogate for such activity. Additionally, it is very challenging to detect a change in a recombinant protein by such an in vivo potency assay, especially when the level of deviation is too small to be detectable by biophysical and biochemical methods which are commonly used for vaccine quality control (QC). To avoid the issue of high variability in in vivo studies and to adhere to the “3R” principle (reduction, replacement and refinement of animals) [9], several in vitro potency assays have been developed as alternatives [10]. ELISA (Enzyme-linked immunosorbent assay)-based in vitro assay is one of them, and such assays have been evaluated both for licensed and developmental vaccines against multiple pathogens, such as human papillomavirus [11], hepatitis B [12], rabies [13], diphtheria [14], hepatitis E [15] and respiratory syncytial virus (RSV) [16].

In the present work, using a well-characterized Pfs230 recombinant protein, Pfs230D1+ [17], and two human anti-Pfs230 monoclonal antibodies (mAbs) which have functional activity measured by SMFA [18], we developed an in vitro sandwich ELISA, called the Antigen Integrity Assay (AIA). The expected usage of this AIA is to demonstrate integrity of functional epitopes in a new lot or a stressed (or stored) protein compared to a reference protein. We evaluated multiple validation parameters (linearity, specificity, intra- and inter-assay precision, and limit of detection) to qualify AIA following International Conference on Harmonization (ICH) Q2(R1) guidelines [19]. To demonstrate such utility, we employed two lots of Pfs230D1+ protein (reference and an engineering lot) together with damaged antigens (reduced-and-alkylated or protease digested). The AIA coupled together with the validation parameters examined, indicate such an assay would be an additional biochemical control measure to incorporate into Pfs230 release and stability campaigns.

## 2. Materials and Methods

### 2.1. Reagents

The details of expression and characterization of the reference Pfs230D1+ recombinant protein have been described elsewhere [17]. In brief, the recombinant protein comprising N-terminal fragment (amino acid (aa) 552–731) of Pfs230 in *P. falciparum* 3D7 strain was produced in a baculovirus expression system. Two human anti-Pfs230 mAbs, Pfs230AS01-18 mAb (denoted as H18 mAb hereafter) and Pfs230AS01-73 mAb (denoted as H73 mAb) were generated based on receptor sequences of antigen-specific B cells isolated from vaccinees who had received Pfs230D1-EPA (a recombinant Pfs230 protein, which contains aa 542–736 of Pfs230 from NF54 strain, conjugated with ExoProtein A) and formulated in GlaxoSmithKline’s AS01 adjuvant (Clinicaltrials.gov # NCT02942277) according to published methods [20]. Both mAbs recognize Pfs230D1+ in a conformation-dependent manner and have functional activities measured by SMFA [18]. As a detection mAb, horseradish peroxidase (HRP) conjugated H18 mAb, H18-HRP mAb, was generated using a HRP Conjugation Kit-Lightning-Link (Abcam, Cambridge, UK. Cat No ab102890) following the manufacturers’ instructions. A mouse anti-Pfs230 mAb, 15A4-1B12, was isolated from mice immunized with Pfs230C1 (aa 443-731) and reacts with Pfs230D1+ protein in a conformation-dependent manner [17], but the mAb does not have SMFA activity. HRP-conjugated 15A4-1B12 mAb was generated as H18-HRP mAb. Bovine Serum Albumin (BSA; Cat No A7906), Tween-20 (Cat No P1379), Dithiothreitol (DTT; Cat No 3483-12-3), Iodoacetamide (IAA; Cat No I1149) and 3.0 kDa Spin Concentrators (Cat No UFC800308) were purchase from Sigma (St. Louis, MO, USA). 1x Phosphate Buffered Saline (PBS; Cat No 10010-023), 2-Mercaptoethanol (BME; Cat No BP176-100) and Slide-A-Lyzer Dialysis Cassettes (Cat No 66330) were obtained from Thermo Fisher (Waltham, MA, USA); Tris buffered saline (TBS; Cat No 351-086-131) from Quality Biological (Gaithersburg, MD, USA); and KPL SureBlue TMB (3, 3’, 5, 5’—Tetramethylbenzidine) Microwell Peroxidase Substrate (TMB substrate; Cat No 5120-0075) and KPL TMB STOP Solution (stop solution; Cat No 5150-0021) from Seracare (Milford, MA, USA).

### 2.2. Antigen Integrity Assay (AIA)

Flat-bottom 96-well ELISA plates (Immulon 4; Thermo Fisher; Cat No 3855) were coated with 200 ng/well of H73 mAb diluted in 1x PBS (pH7.4). The plates were wrapped in plastic wrap and stored at 4 °C overnight. On the next day, H73 mAb solution was discarded, and the plates were blocked with 350 µL/well of blocking/dilution buffer (1xPBS with 1% Casein; BioRad, Hercules, CA, USA; Cat No 1610783) for one hour at room temperature (RT). After extensive washing with washing buffer (0.01% Tween-20 in TBS), an indicated amount of test and reference proteins diluted in blocking/dilution buffer were added to the plates (100 µL/well). Unless otherwise stated, test samples were applied on the ELISA plates at two-fold serial dilutions (a total of six different concentrations) in triplicate wells, and a total of 11 concentrations of two-fold serial dilutions in duplicate wells for the reference protein. In each plate, two wells received only blocking/dilution buffer (blank wells). After 1.5 h of incubation at RT, the plates were washed, and further incubated for 1.5 h at RT with 27.7 ng/well (100 µL/well) of H18-HRP mAb. After washing, 100 µL/well of TMB substrate was added for 10 min at RT in the dark, and the reactions were terminated by adding 100 µL/well of stop solution. Immediately after this addition, the absorbance at 450 nm was read using a VersaMax plate reader (Molecular Devices, Sunnyvale, CA, USA).

### 2.3. Conversion from Absorbance Values to Protein Concentrations for Test Samples, and Calculation of Pfs230D1+ Ratio

For each ELISA plate, the relation between protein concentration (log-transformed) of reference protein and optical density at 450 nm (OD_450_) at 11 different dilutions was approximated by a four-parameter sigmoid fit (Prism 9, GraphPad Software, La Jolla, CA, USA) and used as a standard curve. Based on the standard curve, intact Pfs230D1+ concentration in each test well in the same plate was calculated from the OD_450_ value (denoted as Obs-conc, i.e., observed Pfs230D1+ concentration). A theoretical Pfs230D1+ concentration (Theo-conc) was calculated based on the original intact Pfs230D1+ concentration in the undiluted test sample and the dilution factor. Finally, the Pfs230D1+ Ratio of a test sample at a given dilution was calculated as: Pfs230D1+ Ratio = (Obs − conc)/(Theo − conc).

### 2.4. Generation of Damaged Pfs230D1+ Proteins

Two types of damaged Pfs230D1+ proteins were generated from the reference protein [17], one was trypsin-treated Pfs230D1+ (Tryp-D1+) and the other was reduced-and-alkylated Pfs230D1+ (R&A-D1+). Tryp-D1+ was made using Immobilized Trypsin (Promega, Madison, WI, USA; Cat No V9012) following the manufacturers’ instruction. The protein concentration of Tryp-D1+ was determined by DS-11 FX+ (DeNovix, Wilmington, DE, USA). The observed concentration of Tryp-D1+ (0.45 mg/mL) was within 5% error from a theoretical concentration (0.47 mg/mL) calculated from the original amount of protein used for the reaction and the final volume of solution. To generate R&A-D1+, 1.49 mL of 1M DDT was mixed with 1 mg of original Pfs230D1+ protein for 30 min at 60 °C, then 41 µL of 1 M IAA was added and incubated for 30 min at 37 °C. The reaction was stopped by adding 0.58 mL of BME. Using Slide-A-Lyzer Dialysis Cassettes (3.5 kDa) and 3.0 kDa Spin Concentrators, the R&A-D1+ sample was buffer exchanged to TBS (pH8.0) and concentrated to the same protein concentration of the original Pfs230D1+ protein (0.67 mg/mL). The protein concentration in the final product was determined by DS-11 FX+.

### 2.5. Generation of an Engineering Lot of Pfs230D1+ Protein

The methods for cloning and construction of Bacmid DNA were identical to those described for the reference Pfs230D1+ protein [17] with the exception of removal of the histidine tag from the sequence. This Bacmid was sequence verified and used to transfect ExpiSf9 cells (Thermo Fisher, Carlsbad, CA, USA; Cat. No A35243) for the generation of recombinant baculovirus stock using BaculoFECTIN II and animal-free transfection medium Oxford reagent (Oxford Expression Technologies, Oxford, UK; Cat. No 300105) following the Bac-to-Bac manual. The baculovirus was amplified by three passages in the ExpiSf9 cells grown in ESF921 medium (Expression Systems, Davis, CA, USA; Cat. No 96-001-01) and used to infect the ExpiSf9 culture in the bioreactor. The ExpiSf9 cells were seeded at 2 × 10^6^ cells/mL in ESF921 medium and MOI (multiplicity of infection) of one was used to infect a pair of 25 L ExpiSf9 wave cultures. At 3–4 days post infection and when the viability of the culture dropped to ~75%, the supernatant was harvested by centrifugation and clarified by 0.22 µm filtration. The clarified supernatant was concentrated and diafiltered using 5 kDa nominal molecular weight cut off membrane in 20 mM Tris buffer, pH 8.0 to achieve a 2.5-fold concentration, and clarified with 0.22 µm filtration. The expressed protein was captured and initially purified through Q-Sepharose FF (GE Healthcare, Uppsala Sweden; code no 17051005) column (8.1 × 9.7 cm). The column was equilibrated, loaded, washed and eluted with the same Tris buffer containing 50, 100, 200 and 1000 mM NaCl in a step gradient. The elution was monitored by UV absorbance, and the collected fractions were monitored by SDS-PAGE (sodium dodecyl sulfate-polyacrylamide gel electrophoresis). The Pfs230D1+ rich fraction (100 mM NaCl) was collected, diluted two-fold and allowed to flow through a MEP HyperCel (Sartorius Stadim, Cergy Saint Christophe, France; P/N 12035) column (5 × 11.9 cm), equilibrated with 50 mM NaCl in 20 mM Tris buffer, pH 8.0 to remove impurities. The Pfs230D1+ was then polished on a Capto DEAE (GE Healthcare; code no 17544310) column (5 × 9.6 cm) through binding and step elution with 40, 50, 60, 80, and 100 mM NaCl in 20 mM Tris buffer, pH 8.0. SDS-PAGE was used to confirm the fractions containing Pfs230D1+ (80mM NaCl). Finally, tangential flow filtration (TFF) was used for salt removal, buffer exchange, and concentration into the final buffer of 20 mM HEPES (4-(2-hydroxyethyl-1-piperazineethanesulfonic acid) buffer,150 mM NaCl, pH 7.4. The Pfs230D1+ at 0.3 mg/mL (as reported by bicinchoninic acid (BCA) assay) was stored frozen at <−60 °C.

### 2.6. Characterization of the Engineering lot of Pfs230D1+ Protein

Characteristics of the engineering lot of protein were evaluated as described for the reference Pfs230D1+ protein [17]. Purity was assessed by SDS-PAGE and reverse phase HPLC (RP-HPLC), percent of monomer by Size Exclusion Chromatography HPLC (SEC-HPLC), integrity by western blot using H73 mAb, and protein concentration by BCA assay. The details of methodologies were described previously [17].

### 2.7. Mouse Immunization Study with Two Batches of Pfs230D1+ Proteins, ELISA and SMFA

A group of three female and three male CD-1 mice (7–9 weeks old; Charles River, Wilmington, MA, USA) were immunized with 0.5 or 5.0 µg/dose of Pfs230D1+ protein (either reference or engineering lots of proteins) formulated with Montanide ISA720 (Seppic, Fairfield, NJ, USA) on days 0 and 21 by intramuscular injection, and the serum samples were collected on day 35. The antibody titers of the collected serum samples were evaluated by ELISA [21] using reference or engineering lots of Pfs230D1+ protein as a coating antigen, and functionality of purified IgGs from the sera was assessed by SMFA [8] as described previously.

### 2.8. Statistical Analysis

To evaluate specificity and linearity of AIA, the correlation between Obs-conc and Theo-conc for each of three types of samples or for a combination of all three was assessed by a linear regression. For intra-assay precision, percent CV (%CV) of Obs-conc in triplicate wells was calculated. For inter-assay precision, Pfs230D1+ Ratio was utilized. For a normality test, the D’Agostino and Pearson test was used. To compare antibody titers (ELISA units) between two protein groups at the same immunization dose, log-transformed ELISA units were analyzed by a Student’s-*t* test. To determine a concordance of log-transformed ELISA units against engineering and reference lots of Pfs230D1+ proteins, the best-fit line was compared with y = x line by F test. For SMFA, % inhibition in oocyst density (%TRA) and *p*-values were calculated using a zero-inflated negative binomial (ZINB) model as described [8].

All statistical tests were performed in Prism 9 or R (version 3.5.3, The R Foundation for Statistical Computing) and *p*-values < 0.05 are considered significant.

## 3. Results

### 3.1. Development of AIA

**Figure 1:**
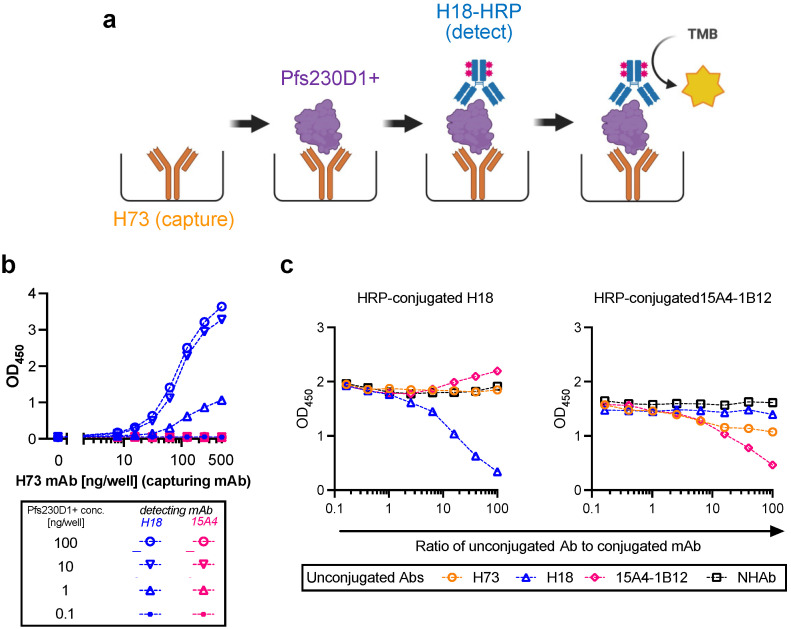
Development of Antigen Integrity Assay (AIA). (**a**) Basic principle of AIA. (**b**) An ELISA plate was coated with H73 mAb at different concentrations (0 to 500 ng/well, *x*-axis), then different concentrations of reference Pfs230D1+ protein (0.1 to 100 ng/well, different shapes) were applied. After wash, a fixed concentration (27.7 ng/well) of either HRP-conjugated H18 (blue) or HRP-conjugated 15A4-1B12 (red, 15A4) mAbs were added to detect the Pfs230D1+ protein. (**c**) Competition ELISA using ELISA plates coated with reference Pfs230D1+ protein. Mixtures of HRP-conjugated H18 (left panel) or HRP-conjugated 15A4-1B12 (right panel) mAbs with unconjugated H73, H18, 15A4-1B12 or normal human antibody (NHAb, as a negative control) were generated at indicated ratio (*x*-axis), and then applied to ELISA plates coated with reference Pfs230D1+ protein (100 ng/well). After 2 h of incubation, the plates were washed, then TMB substrate was added. The OD_450_ signals from HRP-conjugated mAbs are shown in *y*-axis.

A sandwich ELISA using two different human anti-Pfs230 mAbs, H73 and H18 mAbs, was developed (Figure 1a). Both mAbs recognize Pfs230D1+ in a conformation-dependent manner and have functional activities measured by SMFA [18], indicating they recognize functional epitopes in the Pfs230 molecule. To establish the assay conditions, an ELISA plate was coated with H73 mAb at different concentrations (0 to 500 ng/well), then different concentrations of reference Pfs230D1+ protein (0.1 to 100 ng/well) were applied. After washing, a fixed concentration (27.7 ng/well) of either HRP conjugated H18 (H18-HRP mAb) or HRP conjugated 15A4-1B12 mAb (a conformation-dependent mouse mAb, but lacking function) were added to detect the reference Pfs230D1+ protein (Figure 1b). When H18-HRP mAb was used as a detecting mAb, as predicted, a higher concentration of capturing mAb (H73) and higher concentration of reference protein gave higher OD_450_ signals. On the other hand, when HRP-conjugated 15A4-1B12 mAb was used as a detecting mAb, no signal was observed. A competition ELISA showed that there was a competition between H73 and 15A4-1B12 mAbs, but not between H73 and H18 mAbs (Figure 1c), indicating the existence of steric hindrance between H73 and 15A4-1B12 mAbs. Based on the results, the assay was set with 200 ng/well of H73 mAb (capturing mAb), 2-fold serial dilutions of reference protein from 25 ng/well (to generate a standard curve in each plate), and 27.7 ng/well of H18-HRP mAb (detecting mAb).

**Figure 2:**
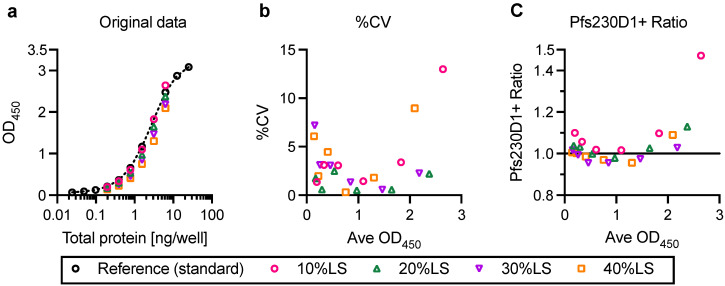
Representative AIA result from a single assay. (**a**) Serial dilutions of reference Pfs230D1+ (no BSA, standard), or 4 different mixtures of Pfs230D1+ and BSA were evaluated by AIA. The mass ratio between Pfs230D1+ and BSA in 10%LS, 20%LS, 30%LS and 40%LS mixtures were 9:1, 8:2, 7:3 and 6:4, respectively. (**b**) Using the standard curve, OD_450_ value in each test well was converted to Pfs230D1+ concentration (Obs-conc). Percent coefficients of variance (%CV) of Obs-conc in triplicate wells for each test sample at each dilution are shown. The *x*-axis shows average OD_450_ value in triplicate wells for the test condition. (**c**) The average Obs-conc in triplicate wells was compared with the corresponding theoretical Pfs230D1+ concentration (Theo-conc) to obtain Pfs230D1+ Ratio.

To determine the analytical format, a sandwich ELISA was performed with mixtures of reference Pfs230D1+ protein and BSA. Four different test samples were made by mixing (1) 90% mass concentration of reference Pfs230D1+ and 10% of BSA (10%LS), (2) 80% and 20% (20%LS), (3) 70% and 30% (30%LS), and (4) 60% and 40% (40%LS). The test samples were applied to H73-coated ELISA plates at 2-fold serial dilutions (0.195 to 6.25 ng/well of total protein) alongside a standard (2-fold serial dilution of reference Pfs230D1+ alone from 0.02 to 25 ng/well). Representative results from a single assay are shown in Figure 2. As expected, 10%LS, 20%LS, 30%LS and 40%LS samples showed lower OD_450_ values than the reference standard at the same total protein concentrations in a dose dependent matter (Figure 2a). Using the standard curve, an OD_450_ value of each test sample at each dilution was converted to Pfs230D1+ concentration, called observed Pfs230D1+ concentration, Obs-conc. Arithmetic mean (average) and %CV of Obs-conc in triplicate wells for each test sample at each dilution were calculated. The average Obs-conc was then compared with the theoretical value, Theo-conc, to determine Pfs230D1+ Ratio. For example, when a 10%LS sample was tested at 6.25 ng/well of total protein, Theo-conc was estimated as 6.25 × 0.9 = 5.625 ng/well. In this case, if the average Obs-conc was 6.2 ng/well, then the Pfs230D1+ ratio was 6.2/5.625 = 1.10. If AIA is a perfect assay (i.e., no error of assay), %CV should be 0 and Pfs230D1+ Ratio should be 1.

CV values were lower than 5% between ~0.5 and ~2.0 OD_450_ range (Figure 2b), and increased at <0.5 OD_450_ or >2 OD_450_. Similarly, Pfs230D1+ Ratios, in general, were closest to 1 in the mid-range of OD_450_ values (Figure 2c), but increased outside of this range, especially when the OD_450_ values were >2. These results suggested that the assay could not accurately determine concentration of intact Pfs230D1+ protein in the test samples when observed OD_450_ values were too high or too low. While test samples were always assessed at six 2-fold dilutions throughout this study, for consistency, Obs-conc of a test sample was calculated from a single dilution (out of 6), which showed the closest OD_450_ value to the midpoint of a sigmoid curve of the standard (usually OD_450_ of ~1.5 to 2) in the following analysis, unless otherwise specified. Three independent AIAs were performed using H18 mAb as a capture mAb and HRP-conjugated H73 mAb as a detection mAb. However, since the results were very similar (data not shown), the following study was performed using only one protocol, where H73 mAb was used as a capture and H18-HRP mAb as a detecting mAb, as written in Materials and Methods.

### 3.2. Qualification of AIA

To qualify the assay, a mixture with BSA (the mixture mimics an imperfect drug substance which is contaminated with unrelated protein), in addition to two more types of imperfect proteins, were utilized. One was a mixture with trypsin-treated Pfs230D1+ (Tryp-D1+; i.e., contaminated with degraded Pfs230D1+), and the other was a mixture with reduced and alkylated Pfs230D1+ (R&A-D1+; i.e., contaminated with non-disulfide bonded Pfs230D1+). SDS-PAGE and western blot (probed with H73 and H18 mAbs) results of Tryp-D1+ and R&A-D1+ are shown in Figure S1. For each type of imperfect protein, 4 different concentrations of mixtures were generated (10%LS, 20%LS, 30%LS and 40%LS), and 6 independent assays were performed. To determine operator-to-operator variability, Tryp-D1+ or R&A-D1+ mixtures were tested by two operators (3 independent assays by each operator, a total of 6 assays).

#### 3.2.1. Linearity and Specificity

**Figure 3:**
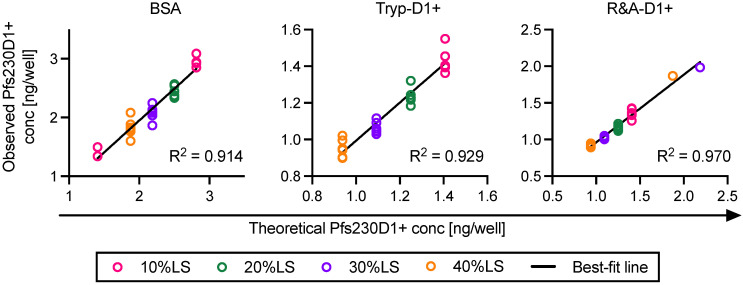
Linearity and specificity of assay. Three types of non-intact Pfs230D1+ proteins (BSA, Tryp-D1+ or R&A-D1+) were mixed with reference Pfs230D1+ at four different ratios (10%LS, 20%LS, 30%LS or 40%LS), then each of the mixtures was tested in six independent AIA. The results from each assay (dot), the best-fit line, and R^2^ for the linear fit are shown. The dilution (out of 6 dilutions tested for each mixture) that gave the closest OD_450_ value to the midpoint of the standard sigmoid curve was not necessarily the same in all assays. Therefore, the theoretical concentrations were different in several assays even when the same mixtures were tested (two assays in 10%LS with BSA, and one assay each in 30%LS and 40%LS with R&A-D1+).

For the specificity and linearity evaluation, theoretical Pfs230D1+ concentration (Theo-conc) and observed concentration (Obs-conc) were compared (Figure 3). For each type of sample, a linear correlation appeared between Theo-conc and Obs-conc (R^2^ > 0.91, evaluation for the linearity of assay). When all data were combined (*n* = 72), R^2^, slope and y-intercept of the best-fit line were calculated as 0.97, 1.00 (95% confidence interval, 95%CI, 0.97 to 1.04) and −0.04 (95%CI; −0.10 to 0.03), respectively, indicating a strong concordance between Theo-conc and Obs-conc. Thus, AIA can reasonably determine the concentration of intact Pfs230D1 + protein in a test sample which includes predicted impurities (specificity of assay).

#### 3.2.2. Intra- and Inter-Assay Precision, and Limit of Detection

**Figure 4:**
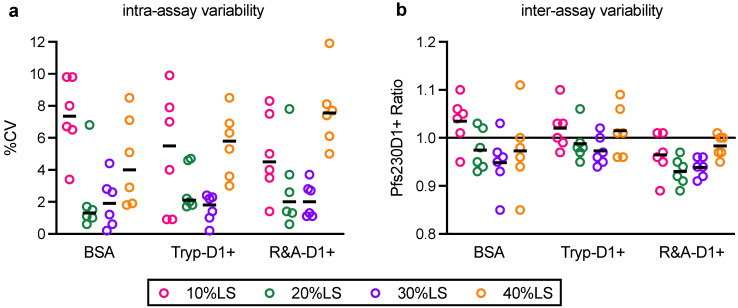
Intra- and inter-assay variability. AIA was performed as described in Figure 3, and %CV in triplicate wells (**a**) and Pfs230D1+ Ratio (**b**) in each assay (dots) are shown. The bars represent median in (**a**) and mean in (**b**). The assays with BSA were performed by a single operator (6 independent assays), while Tryp-D1+ and R&A-D1+ were tested by two operators (3 independent assays per operator).

Using the same data set, intra- and inter-assay variabilities were evaluated. For the intra-assay variability, %CV of Obs-conc in triplicate wells were <10%, except one (%CV of 11.9, 40%LS with R&A-D1+) out of 72 data points (Figure 4a). The %CV data did not pass a normality test (*p* = 0.026), and the median %CV was 3.2 (25 and 75 percentiles of 1.4 and 6.8, respectively). For the inter-assay variability (Figure 4b), a majority (66 out of 72) of Pfs230D1+ Ratio was within the range between 0.9 to 1.1 (+/- 10% error). The Pfs230D1+ Ratio passed a normality test (*p* = 0.367), indicating the variation in Pfs230D1+ Ratio was likely to be a random error. The mean, standard deviation (sd) and %CV of Pfs230D1+ Ratio were calculated as 0.979, 0.053 and 5.4, respectively. Therefore, if an observed Pfs230D1+ Ratio of a test sample is less than 0.88 (mean-1.96 x sd), with >95% probability, the sample is truly worse than the reference Pfs230D1+ protein (limit of detection).

In addition to the total inter-assay variability, operator-to-operator variability in Pfs230D1+ Ratio was assessed using Tryp-D1+ and R&A-D1+ data where each of two different operators (Ope 1 and Ope 2) performed three independent assays (Figure S2). To determine operator-to-operator variability, %CV for each “paired” data set was calculated. “Paired” in this analysis meant that Obs-conc (average of triplicate wells) of the 1st assay with Trpy-D1+ at 10%LS by Ope 1 was compared to that of the 1st assay with the same sample by Ope 2. Obs-conc of the 2nd assay with R&A-D1+ at 20%LS by Ope 1 was compared to that of the 2nd assay with the same sample by Ope 2, and so on. A total of 24 different %CV values were calculated from the 24 “paired” data sets (2 types of samples were tested at 4 different mix ratios in 3 independent assays). The %CV values did not pass a normality test (*p* = 0.002), and the median %CV was 3.0 (25 and 75 percentiles of 1.2 and 3.7, respectively) with maximum of 9.1%.

Taken together, our results determined that (1) intra-assay precision of AIA is 3.2%CV, (2) total inter-assay precision is 5.4%CV, operator-to-operator precision (a part of inter-assay precision) is 3.0%CV, and (3) limit of detection is 12% loss (Pfs230D1+ Ratio of <0.88) of intact Pfs230D1+ protein in a test material. The summary of assay qualification is shown in Table 1.

#### 3.2.3. Robustness of Assay

**Figure 5:**
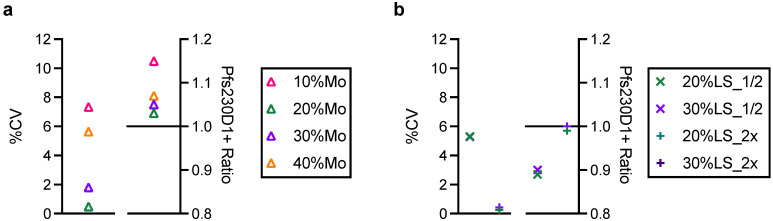
Robustness of assay. (**a**) AIA was performed with 10%, 20%, 30% or 40% more reference Pfs230D1+ proteins (10%Mo, 20%Mo, 30%Mo or 40%Mo, respectively) in test wells. Left-side panel shows %CV in triplicate wells and right-side panel for Pfs230D1+ Ratio. (**b**) AIA was performed with half (20%LS_1/2 or 30%LS_1/2) or double (20%LS_2x or 30%LS_2x) concentration of H18-HRP mAb using 20%LS and 30%LS samples with BSA. %CV for 20%LS_1/2 and 30%LS_1/2 were both 5.3, thus only a single symbol is seen in Figure 5b.

In anticipation of the future full validation of AIA to support subsequent cGMP (current Good Manufacturing Practice) of the Pfs230D1+ protein, two aspects for the robustness of the assay were evaluated. As described before, the expected usage of AIA is to prove a non-inferiority of integrity in a new lot of protein to a reference protein. Thus, the assay development and qualification were performed with reference protein mixed with BSA or damaged proteins. In other words, all test samples were worse (as in purity, concentration, or integrity) than the reference. However, hypothetically, a new lot of protein could be superior to that of the reference protein. Therefore, AIA was performed with 10/20/30/40% more reference proteins, instead of 10/20/30/40% less reference proteins (Figure 5a). The %CV values were <7.3%, and Pfs230D1+ Ratios were between 1.03 to 1.15, indicating that the AIA is not likely to give a false positive result (i.e., <0.88 Pfs230D1+ Ratio), when a test sample includes more intact protein.

A second robustness test was done to evaluate the impact of H18-HRP mAb concentration, because HRP-conjugation efficiency could vary among different batches of H18-HRP mAbs. To test this, AIA was performed with half (13.9 ng/well) or double (55.4 ng/well) concentration of H18-HRP using 20%LS and 30%LS samples with BSA (Figure 5b). The %CV was <5.3%, and Pfs230D1+ Ratio was between 0.89 to 1.00, indicating the AIA is robust to the change in the H18-HRP mAb concentration.

### 3.3. Quality Control (QC) of AIA

**Figure 6:**
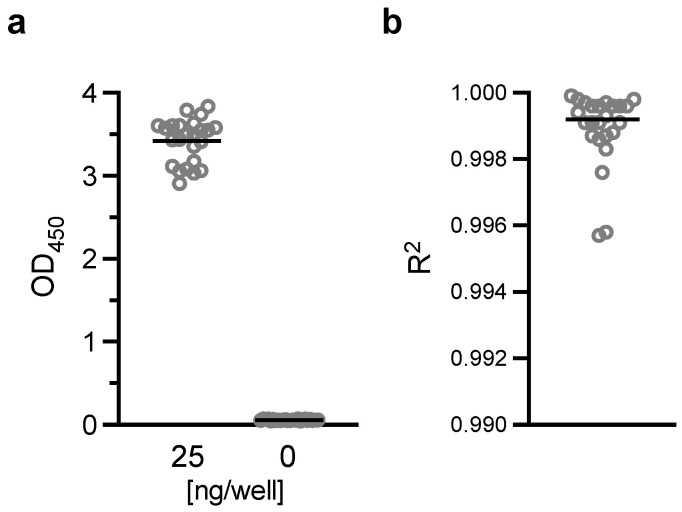
Quality control for standard. (**a**) AIA was performed as described in Figure 3. OD_450_ values at the highest (25 ng/well) or the lowest (0 ng/well, blank) concentrations (**a**), and R^2^ of 4-parameter sigmoid fits (**b**) of standards from the 24 independent assays are shown (dots). The bars represent mean in (**a**) and median in (**b**).

Obs-conc (and resulting Pfs230D1+ Ratio) of a test sample is calculated using a standard curve in the same plate, thus, establishing criteria for QC of the standard is important. To this end, results from the 24 independent assays, where data for Figure 3 and Figure 4 were generated, OD_450_ at the highest (25 ng/well of reference protein) and the lowest (0 ng/mL, blank wells) concentrations, and R^2^ values (for the four-parameter sigmoid fit) were analyzed (Figure 6). The OD_450_ values passed normality tests (*p* > 0.300), and the mean (sd; range) was 3.420 (0.264; 2.907–3.836) and 0.058 (0.007; 0.046–0.070) at 25 and 0 ng/well, respectively. The R^2^ data did not pass a normality test (*p* < 0.0001) and median (range) of 0.999 (0.996–1.000). Based on the results, we define an assay as valid if all following criteria are met for the standard; (1) OD_450_ value at 25 ng/well is >2.800, and <0.080 at 0 ng/well, and (2) R^2^ > 0.994.

Similarly, based on Figure 4a data, we define QC criterion for a test sample in an assay as %CV in triplicate wells should be <12.

### 3.4. Evaluation of an Engineering lot of Pfs230D1+ (Eng) Protein

**Figure 7:**
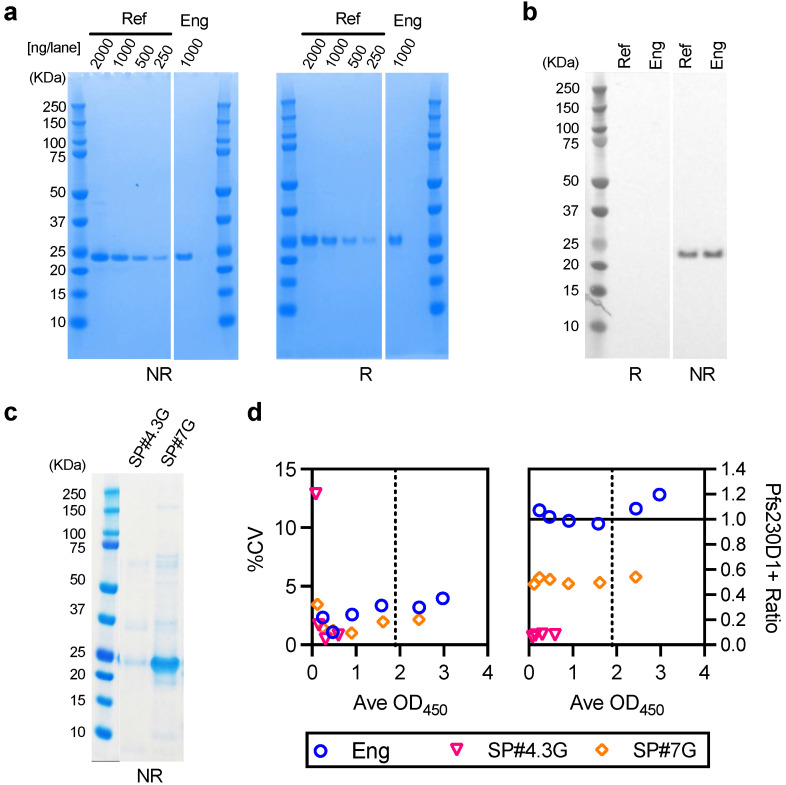
Evaluation for the engineering lot of Pfs230D1+ (Eng). Reference (Ref) and engineering (Eng) lot of Pfs230D1+ proteins were evaluated by SDS-PAGE (**a**) and western blot (**b**) both under reducing (R) and non-reducing (NR) conditions. The western blot was done with H73 mAb. (**c**) two in-processed proteins, SP#4.3G and SP#7G were tested by SDS-PAGE under a non-reducing condition. Uncropped SDS-PAGE and western blot images are seen in Figure S3. (**d**) AIA was performed with Eng, SP#4.3G and SP#7G proteins. Since OD_450_ values for SP#4.3G did not reach to the midpoint of standard sigmoid curve (vertical dotted line, OD_450_ = 1.9), %CV and Pfs230D1+ Ratio at all 6 dilutions for each test sample are shown.

The reference protein had been previously made in a Research & Development (R&D) setting but moving forward to future cGMP production, in this study an engineering lot of Pfs230D1+ protein (Eng) was produced at the 50L scale using a Baculovirus expression system and the final intended cGMP manufacturing process. The supernatant was harvested 3–4 days post infection and purified by column chromatography. There was no detectable difference between reference and Eng proteins by SDS-PAGE and western blot analysis (Figure 7a,b) utilizing the established release assays. Based on Size Exclusion Chromatography HPLC (SEC-HPLC), Eng protein was 100% monomer, and 96.8% pure under reduced and 95.8% pure under non-reduced conditions by Reverse Phase HPLC (RF-HPLC; Figure S3).

Two in-process samples were evaluated by SDS-PAGE; SP#4.3G and SP#7G corresponding to the 50 and 100 mM NaCl in-process fractions from the Q-Sepharose column purification. The SP#4.3G was not included in the Q-Elution pool, however the SP#7G fraction was further purified using MEP HyperCel and Capto DEAE columns to generate the final Eng protein as described in Materials and Methods. On SDS-PAGE, while it was clear the SP#7G fraction contained more total proteins, comparing purity between SP#4.3G and SP#7G was difficult from Figure 7c results; SP#4.3G bands were very weak, while the main band for SP#7G could be oversaturated. To test their integrity, and to inform whether the SP#4.3G fraction can be added to future purification pools, an AIA was performed using these two in-process samples to determine if there were any appreciable differences in conformation. The AIA passed all QC criteria for the standard (OD_450_ = 3.373 and 0.0060 at 25 and 0 ng/well, and R^2^ = 0.999), and %CV was lower than <12% for test sample triplicates, except SP#4.3G when tested at the lowest concentration (%CV = 12.9 at average OD_450_ = 0.083). As shown in Figure 7d, for SP#4.3G, even at the highest concentration tested (i.e., a total protein of 6.25 ng/well), the OD_450_ value was much lower (0.596) than the midpoint of the standard sigmoid curve (OD_450_ = 1.900). Therefore, SP#4.3G and SP#7G cannot be compared quantitively. However, Pfs230D1+ Ratio of SP#4.3G was much lower than that of SP#7G at all dilutions, indicating that SP#4.3G was not as pure as SP#7G.

In the same AIA, Eng (final purified) protein was also evaluated, and the %CV of 3.4 and Pfs230D1+ Ratio was 0.96 at the dilution, which gave the closest OD_450_ value to the midpoint of sigmoid curve of the standard. The results suggested that Eng protein would have the same potency as the reference protein.

**Figure 8:**
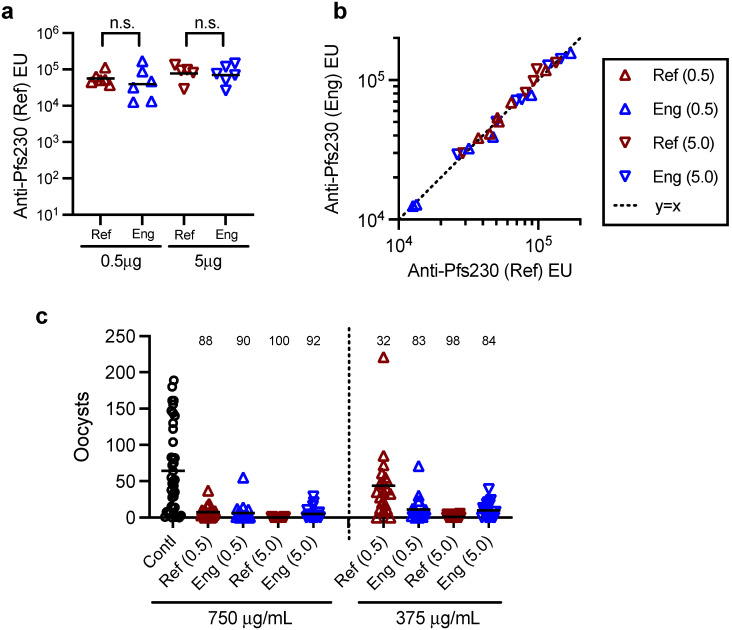
Evaluation for the engineering lot of Pfs230D1+ (Eng). A group of 6 CD-1 mice were immunized with 0.5 or 5 μg/dose of reference (Ref) or engineering (Eng) lot of Pfs230D1+ protein on days 0 and 21, and serum samples were collected on day 35. (**a**) Anti-Pfs230D1+ ELISA units (EU) against reference protein (Anti-Pfs230 (Ref) EU) were determined by ELISA, and individual (dots) and geometric mean (bars) EU are shown. n.s., not significant (**b**) The same antisera were also tested by ELISA against engineering lot of protein (Anti-Pfs230 (Eng) EU). Anti-Pfs230 (Ref) EU and anti-Pfs230 (Eng) EU of individual sera are shown with a y = x line. (**c**) For each group, total IgG was purified from pooled day 35 sera, and tested by standard membrane-feeding assay at 750 and 375 μg/mL in the presence of complement. As a control (Contl), a total IgG purified from normal mouse sera was tested at 750 μg/mL, and oocyst data in individual mosquitoes are shown with an average (bar) for each group. Percent inhibition in oocyst density (%TRA) was calculated against Contl group, and shown for each group at each concentration.

To confirm functional immunogenicity of Eng protein, a mouse immunization study was conducted using the reference protein as a control. Mice were immunized with either 0.5 or 5.0 µg doses on days 0 and 21, and then day 35 sera were collected for evaluation. At each immunization dose, ELISA units did not differ between immunogens based on reactivity to the reference protein (Figure 8a, *p* > 0.451). However, as predicted, %CV in ELISA units within groups were high (ranging from 44 to 100%), and the large animal-to-animal variation within groups might mask any immunological difference between the two proteins. Therefore, the sera were further tested by ELISA for reactivity to Eng protein (Figure 8b). For each immunogen (combined data at two different doses), the best-fit line was not different from the y = x line (*p* = 0.509 and 0.302 for Eng and reference protein immunized groups, respectively). Thus, the two Pfs230D1+ proteins were not immunologically distinguishable based on the ELISA reactivity of the mouse sera. Finally, functional activity of the induced antibodies was determined by SMFA at 750 and 375 µg/mL in the presence of human complement. Except for the IgG from mice immunized with 0.5 µg dose of reference protein and tested at 375 µg/mL (32% inhibition in oocyst density, *p* = 0.313), all IgGs showed > 2% inhibition (*p* < 0.001). The results confirmed the Eng protein induces functional antibodies in mice, consistent with the reported AIA for the engineering lot.

## 4. Discussion

In this study, using two human functional mAbs, we developed an in vitro potency assay, AIA, against the lead malaria transmission blocking vaccine candidate, Pfs230D1+. As described before, there are multiple reports for the development of in vitro ELISA-based potency assays against other pathogens. However, to the best of our knowledge, this is the first report for malaria vaccines. We qualified the assay following International Conference on Harmonization (ICH) Q2(R1) guidelines [19], while the majority of other studies did not qualify or validate the assay, with few exceptions [22]. While a full validation of an assay is not required until a Phase 3 trial, detailed evaluation of the assay, such as precision and limit of detection, is critical to correctly assess whether a new lot or a stressed protein (or vaccine formulation) is truly different from a reference (original) one, or a difference seen in an assay readout is within the error of assay. These results serve as a basis to incorporate the AIA into future release and stability programs related to the Pfs230D1+ protein.

In this study, linearity, specificity, intra- and inter-assay precision, and limit of detection of AIA were evaluated. Based on the Food and Drug Administration (FDA) guidance [23], intra- and inter-assay precision is recommended to be <20%CV, and AIA passed the criteria (%CV of intra- and inter-assay precision was determined as 3.2 and 5.4, respectively). However, several assay validation parameters were not assessed in this study (Table 1), such as accuracy and inter-laboratory precision. The accuracy was not evaluated here because there is no field-accepted control reagent or method to determine the “true” value. For inter-laboratory precision, there is no plan to evaluate potency of the specific Pfs230 recombinant protein, which was used in this study, in multiple laboratories. Further qualification or full validation of AIA is anticipated when the Pfs230D1+ vaccine moves to phase 2 and 3 trials. In this study, a recombinant protein (drug substance) was used as a test material, but the final vaccine formulation (drug product) will likely include both the protein and an adjuvant(s) to induce strong and durable responses in humans. Thus, once an adjuvant system is selected for a human clinical trial, the utility of AIA for the drug product will be evaluated.

Practically speaking, in vivo animal immunization studies are unavoidable during novel vaccine development (e.g., testing a new antigen, new vaccine formulation and/or new delivery system). However, using such in vivo assays for quality control, process development, stability testing, or monitoring the lot-to-lot consistency of recombinant protein (or vaccine formulation) is not necessarily the best choice, because in vivo immunization assays can be highly variable and are usually time-consuming. Therefore, for those purposes, an in vitro assay is preferable to an in vivo assay where possible. US and European regulatory agencies have accepted use of ELISA-based in vitro potency assays for hepatitis B and human papillomavirus vaccines [6].

Once a surrogate in vitro assay is developed, it is ideal to prove the correlation between in vivo and in vitro potency readouts. However, the poor precision of in vivo assays makes this difficult. When an in vitro potency assay was tested for a human papillomavirus vaccine, there was a linear correlation between in vivo and in vitro assay readouts with the correlation coefficient value of 0.75 [11]. However, the study involved a severely damaged vaccine (the vaccine formulation was treated with DTT and it resulted in >10-fold difference in median in vivo potency between intact and damaged vaccine groups) and the analysis was done using data from >3600 mice (80 mice per assay, and >45 independent in vivo studies). In another study for respiratory syncytial virus vaccine, where a smaller number of animals (*n* = 10 mice per group) was evaluated, the correlation between in vivo and in vitro readouts was weak (the correlation coefficient value or *p*-value was not reported in the manuscript) [16]. In this study, we performed an in vivo animal immunization study to test whether the Eng protein could induce functional antibodies in mice, as seen with the reference protein. Our intention was not to check whether the two proteins were equivalent in functional immunogenicity, or whether there was a correlation between in vitro and in vivo potency assay readouts. As shown in this study, the difference in geometric mean ELISA units between 0.5 and 5.0 µg dose groups of the same protein was smaller than the animal-to-animal variation within the group. In other words, the in vivo study design did not allow detection of a 10-fold difference in “intact” protein concentration. A huge study with a severely damaged protein is likely to be required if one wants to prove the correlation between the two assay readouts.

To assess the utility of AIA as a stability test, reference Pfs230D1+ protein was incubated at 37 °C for 7 days (37C-7D-D1+) and evaluated by AIA. The 37C-7D-D1+ protein was mixed with intact protein, and tested at 10, 20, 30 and 40%LS conditions, as tested with two types of damaged proteins (Tryp-D1+ and R&A-D1+). Surprisingly, all 10, 20, 30 and 40%LS samples with 37C-7D-D1+ demonstrated the same OD_450_ values as reference protein alone (standard) at the same total protein concentration in AIA (Figure S4), indicating no change after the incubation. Subsequent SDS-PAGE and western blot analysis also showed that there was no detectable difference between the original and 37C-7D-D1+ proteins (Figure S1). While the results were encouraging for future TBV development using this recombinant protein, a further study is required before applying the AIA as a part of stability tests.

One of the limitations of this study was assay qualification was performed only up to 40% mixture of imperfect samples. Therefore, the AIA was not evaluated whether the linearity holds true at all ranges of impurity. For example, Pfs230D1+ Ratio of SP#4.3G and SP#7G were ~0.1 and ~0.5, respectively, but we cannot conclude that those samples contained ~10% and ~50% of intact Pfs230D1+ protein. Again, the expected usage of AIA is to prove the non-inferiority of integrity in a test protein compared to a reference protein, thus we believe our approach was sufficient. If a protein is severely damaged (i.e., >40% of protein is damaged), such change will be easily detected by routine SDS-PAGE, western blot or other typical protein QC analyses. On the other hand, if this AIA is to be used to determine a concentration of intact protein in a test material (at all ranges), then additional assay qualification is required. A second limitation here is that in theory, this assay only evaluates integrity of two different functional epitopes detected by the two mAbs, while it is likely that the Pfs230D1+ protein (and other Pfs230-based current vaccine candidates) contains more than two functional epitopes. As generation of human mAbs from vaccinated volunteers from phase 1 trials (or humanized mice) is becoming popular, once new functional, non-competing, mAbs are identified, such mAbs can be quickly applied to the AIA. A single operator can easily test up to 16 different protein samples in two days (including time of coating ELISA plates). Thus, even if the AIA needs to be done with 5–10 different combinations of mAbs, compared to the traditional in vivo potency assay, AIA can provide much higher throughput with excellent sensitivity and precision as described here.

Finally, the AIA could be incorporated into “point of use” stability studies, where the Pfs230 integrity (and presentation of pertinent epitopes) is monitored in the presence of adjuvants and used to support clinical pharmacy manipulations. Given sequence similarity of Pfs230D1+ to other Pfs230 vaccine candidates, such as Pfs230D1M [24], the assay could directly be applied. General methodologies and approach to the assay validation described herein are amenable to any subunit vaccine as long as more than two functional, non-competing, mAbs are available. Thus, this study strongly supports future subunit vaccine development and use of in vitro assays for release and stability of recombinant vaccine candidates.

## Figures and Tables

**Table 1:** Assay characteristics.

Validation Parameter ^1^	AIA ^2^	Results
Specificity	Whether we can detect intact Pfs230D1+ protein in the presence of impurity which may be expected to be present in a test sample	Yes
Linearity	Whether Obs-conc is directly proportional to the Theo-conc.	R^2^ = 0.97 in a linear regression
Range	The interval between the upper and lower levels of impurity of samples where the analytical procedure has a suitable level of Precision and Linearity	From 12% up to (at least) 40% impurity
Accuracy	Agreement between a conventional true value and an observed Pfs230D1+ Ratio	Not determined
Precision		
Repeatability	Intra-assay variability	3.2%CV
Intermediated Precision	Inter-assay variability	5.4%CV
Reproducibility	Inter-laboratory variability	Not determined
Detection Limit	The lowest impurity can be detected	12%
Quantitation Limit	The lowest impurity can be quantitatively detected	Not applicable for the usage of assay

^1^ Seven parameters of assay validation in the International Conference on Harmonization (ICH) Q2(R1) guidelines. ^2^ Specific measurement in the context of AIA.

## Data Availability

The data that support the findings of this study are available from the corresponding author upon request. The uncropped SDS-PAGE and western blot images for Figure 7 are seen in Figure S3.

## Supplementary Materials

The following supporting information can be downloaded at: https://www.mdpi.com/article/10.3390/vaccines10101628/s1, Figure S1: SDS-PAGE and western blot analysis for damaged Pfs230D1+ proteins; Figure S2: Operator-to-operator variability in AIA; Figure S3: Characterization of engineering lot of Pfs230D1+ protein; Figure S4: AIA with 37C-7D-D1+ protein.
