# Supplementary material for: Development and Qualification of an Antigen Integrity Assay for a Plasmodium falciparum Malaria Transmission Blocking Vaccine Candidate, Pfs230
Source: Vaccines (Basel). 2022 Sep 28;10(10):1628. doi: 10.3390/vaccines10101628 (PMC9607959; doi:10.3390/vaccines10101628)
Supplement: Supplementary file 1 [file vaccines-10-01628-s001.zip › vaccines-1941025-supplementary.pdf]

SUPPLEMENTAL MATERIAL

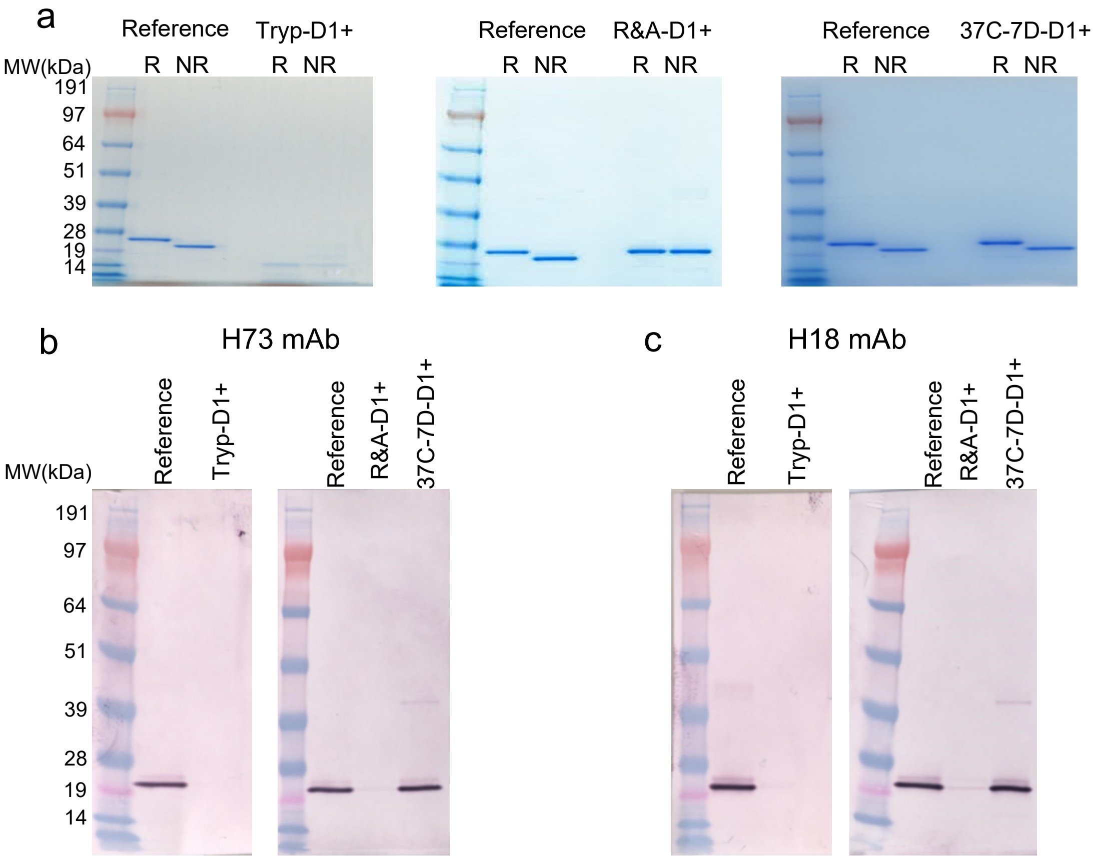

Figure S1. SDS-PAGE and western blot analysis for damaged Pfs230D1+ proteins. (**a**) Three types of damaged proteins were tested with (original) reference Pfs230D1+ protein by SDS-PAGE both under reducing (R) and non-reducing (NR) conditions. Tryp-D1+, trypsin-treated Pfs230D1+ protein; R&A-D1+, reduced and alkylated Pfs230D1+; 37C-7D-D1+, the reference protein was incubated at 37 °C for 7 days. The same proteins were also assessed by western blot using H73 (**b**) or H18 (**c**) mAbs under a non-reducing condition.

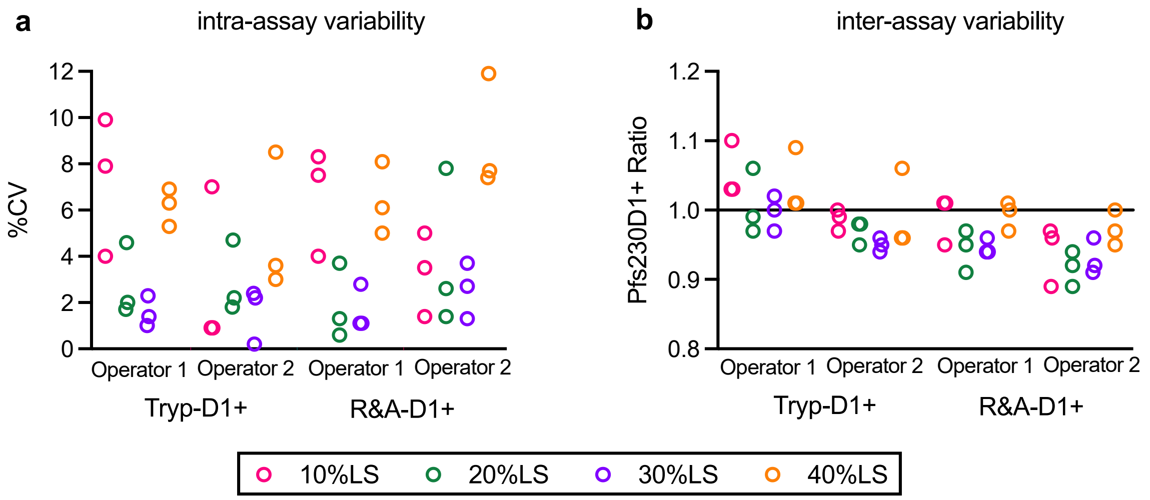

Figure S2. Operator-to-operator variability in AIA. Tryp-D1+ and R&A-D1+ results shown in Figure 4 are separated by operator. The %CV in triplicate wells (**a**) and Pfs230D1+ Ratio (**b**) in each assay (dots) are shown.

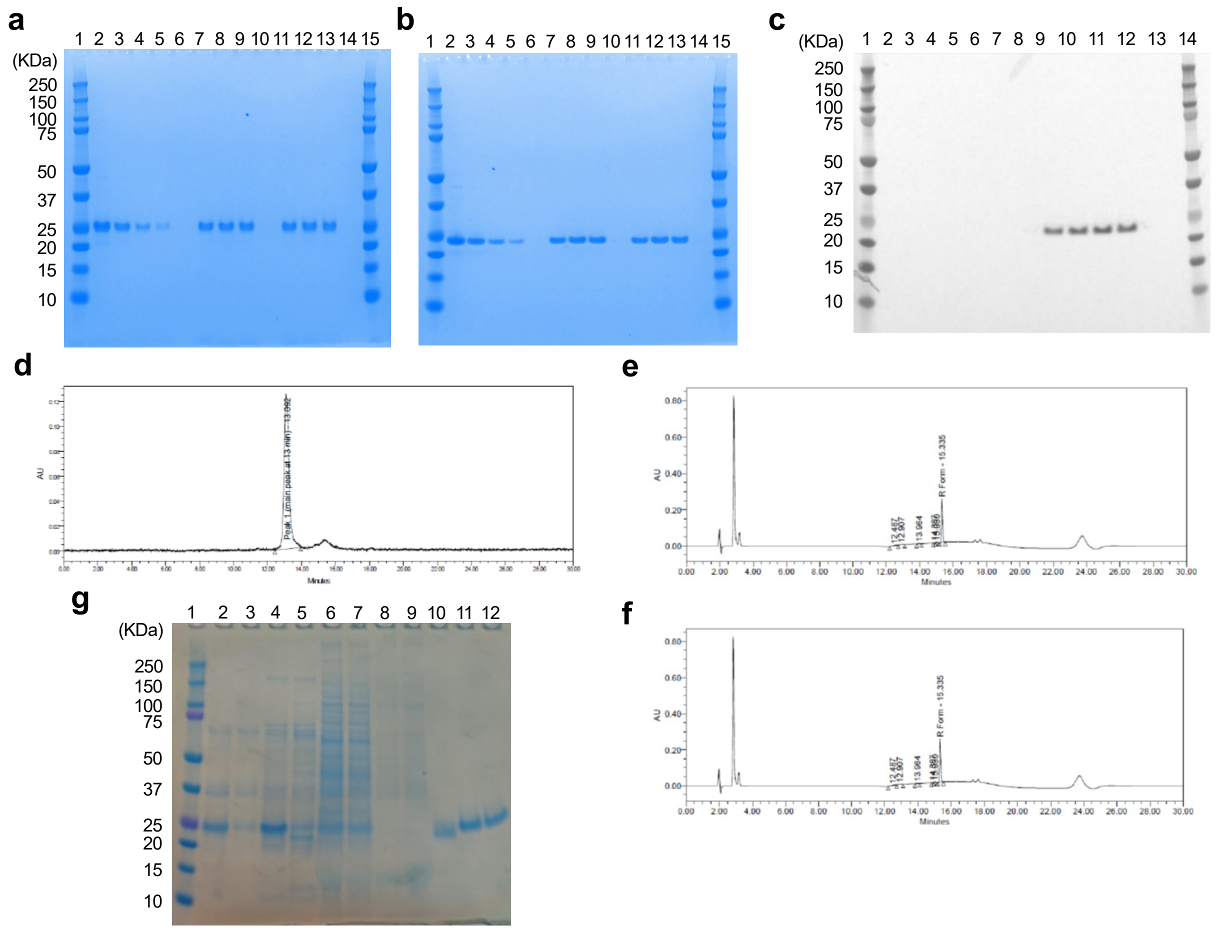

Figure S3. Characterization of engineering lot of Pfs230D1+ protein. Reference (Ref) and engineering (Eng) Pfs230D1+ proteins were evaluated by SDS-PAGE both under reducing (**a**) and non-reducing (**b**) conditions. Lanes 1 and 15; molecular weight standards. Lanes 2-5; Ref at 2000, 1000, 500 and 250 ng/well. Lanes 6, 10, and 14; blank lanes. Lanes 7-9; Ref at 1000 ng/well. Lanes 11-13; Eng at 1000 ng/well. Lanes 1-5 and 13-15 are shown in Figure 7a as a composite image. (**c**) Western blot analysis for Ref and Eng proteins. Lanes 1 and 14: molecular weight standard. Lanes 2-5: reducing and lanes 9-12, non-reducing condition. Lanes 6-8 and 13; blank. Lanes 2 and 9 are Ref, lanes 3-5 and 10-12 are Eng. The loading amount was 50 ng/well and detected by H73 mAb. Lanes 1-3, 9 and 10 are shown in Figure 7b as a composite image. Eng protein was analyzed by SEC-HPLC (**d**), and RP-HPLC under non-reducing (**e**) and reducing (**f**) conditions. (**g**) In-process Pfs230D1+ protein samples from Q-sepharose purification, including SP#4.3G (lane 3) and SP#7G (lane 4) were tested by SDS-PAGE under reducing

condition. Lane 1; molecular weight standard. Lanes 2; Q-Sepharose elution from a different lot of Pfs230D1+ produced (Technology Transfer Lot) and not discussed in this paper. Lane 3 and Lane 4; Q-Sepharose (Eng) elutions at 50 mM and 100 mM NaCl respectively. Lanes 5; Technology transfer lot 100 mM NaCl elution (not discussed). Lanes 6; Technology transfer lot Q-Sepharose Load (not discussed). Lane 7; Q-Sepharose load (Eng). Lane 8; Q-Sepharose Flow-through. Lane 9; Technology transfer lot S-Sepharose flow-through. Lanes 10-11; Technology transfer lot final purified protein loaded at 3 μg/well. Lane 12; (Ref) final purified protein loaded at 3 μg /well. Lanes 1, 3 and 4 are shown in Figure 7c as a composite image.

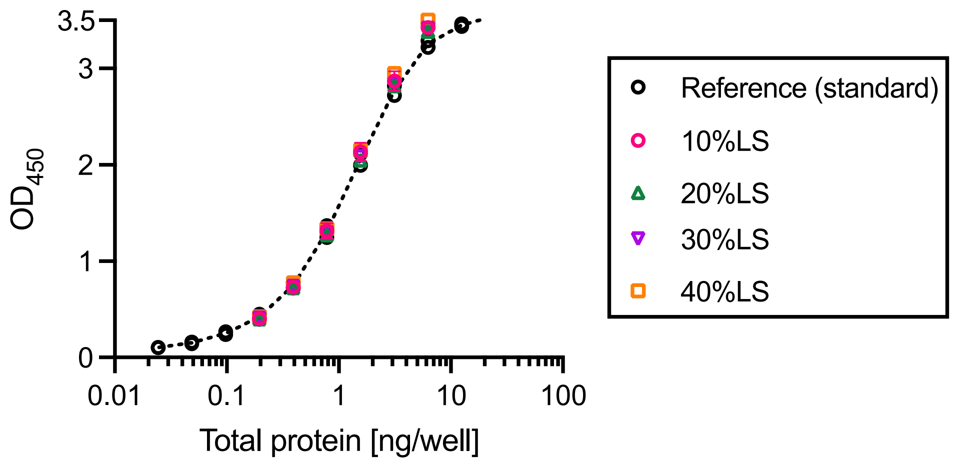

Figure S4. AIA with 37C-7D-D1+ protein. Reference Pfs230D1+ protein was incubated at 37 °C for 7 days (37C-D7-D1+). The 37C-D7-D1+ protein was mixed with reference protein at four different ratios (10%LS, 20%LS, 30%LS or 40%LS) and tested by AIA, as Figure 2.
